# A 12-Week Structured Antioxidant-Focused Dietary Intervention Improves Cognitive Function and Oxidative Stress Biomarkers in Lung Cancer Patients with Cancer-Related Cognitive Impairment: A Randomized Controlled Trial

**DOI:** 10.3390/antiox15080932

**Published:** 2026-07-28

**Authors:** Zhenzhen Huang, Xinxin Cheng, Jianyun He, Lan Cheng, Yuting Wang, Xiaoxia Lin, Xinyi Miao, Ran Wang, Shufang Xia

**Affiliations:** Wuxi School of Medicine, Jiangnan University, Wuxi 214122, China; huangzhenzhen@stu.jiangnan.edu.cn (Z.H.); chengxinxin@stu.jiangnan.edu.cn (X.C.); hejianyun@stu.jiangnan.edu.cn (J.H.); chenglan@stu.jiangnan.edu.cn (L.C.); wangyuting@stu.jiangnan.edu.cn (Y.W.); linxiaoxia@stu.jiangnan.edu.cn (X.L.); miaoxinyi@stu.jiangnan.edu.cn (X.M.); wangran@stu.jiangnan.edu.cn (R.W.)

**Keywords:** Montreal Cognitive Assessment, dietary oxidative balance score, oxidative stress, glutathione, quality of life

## Abstract

Patients with lung cancer are at increased risk of developing cancer-related cognitive impairment (CRCI), and oxidative stress may contribute to its development. We conducted an assessor-blinded randomized controlled trial to evaluate whether a 12-week antioxidant-focused dietary intervention improved cognitive function, oxidative stress biomarkers, and quality of life (QoL) in lung cancer patients with CRCI. One hundred participants were randomized to standard dietary counseling or an antioxidant-focused dietary intervention. Continuous outcomes were assessed at baseline and week 12 and analyzed using linear mixed-effects models under the intention-to-treat principle. The mean age was 66.10 ± 5.39 years, and 78.0% of participants were male. Ninety-one participants completed the week-12 assessment. Participants receiving the intervention demonstrated greater increases in Montreal Cognitive Assessment scores (*β* = 2.626; 95% CI: 0.825, 4.427; *p* = 0.005), dietary oxidative balance score (*β* = 2.292; 95% CI: 0.656, 3.928; *p* = 0.006), and physical and mental QoL component scores than those in the control group (*p* < 0.05). In the complete-case analysis (*n* = 91), the proportion meeting CRCI criteria was lower in the intervention group (60.0% vs. 93.5%; *p* < 0.001). Biomarker analyses showed increases in glutathione and superoxide dismutase and a reduction in malondialdehyde in the intervention group (*p* < 0.05). The intervention demonstrated a large effect on cognitive function (Cohen’s *d* = 0.84), supporting antioxidant-focused dietary intervention as a promising supportive-care strategy for managing CRCI in lung cancer patients.

## 1. Introduction

Lung cancer continues to impose a substantial global health challenge, accounting for more than 2 million newly diagnosed cases and nearly 1.8 million deaths annually worldwide [1]. Despite advances in treatment, patients often experience substantial physical and psychological burden. Cancer-related cognitive impairment (CRCI) is increasingly acknowledged as an important clinical issue among patients with cancer, with reported prevalence estimates ranging from 6% to 84.4% [2]. CRCI is characterized by deficits in working or short-term memory, attention, executive function, orientation, language comprehension, and processing speed, which may persist long after treatment completion and adversely affect daily functioning, treatment adherence, and quality of life (QoL) [2,3]. Despite its clinical significance, effective strategies for the management of CRCI in lung cancer patients remain limited.

Current interventions for CRCI include both pharmacological and non-pharmacological approaches. However, pharmacological therapies have shown inconsistent efficacy and may be associated with adverse neurotoxicity or impaired brain plasticity [4]. Consequently, increasing attention has been directed toward lifestyle-based non-pharmacological interventions because of their safety, feasibility, and potential for long-term implementation [5,6]. Among these, dietary interventions have attracted growing interest, as diet represents a modifiable factor closely associated with cognitive health. Epidemiological evidence has suggested that unhealthy dietary patterns are related to increased risk of cognitive impairment [7], whereas greater adherence to healthy dietary patterns is associated with increased cognitive function and decreased cognitive decline [8]. However, evidence regarding the effectiveness of dietary interventions for established CRCI in cancer populations remains limited.

A variety of studies suggest that oxidative stress is an important contributor to the development of CRCI [9,10]. Cancer progression and anticancer treatments can produce excessive reactive oxygen species (ROS), damage endogenous antioxidant defense systems, and promote neuroinflammation, mitochondrial dysfunction, and neuronal injury, ultimately leading to cognitive decline [11,12]. Dietary antioxidants, including vitamins, carotenoids, and polyphenols, may help counteract these processes by modulating systemic redox homeostasis and reducing oxidative damage [13,14]. A study of postmenopausal breast cancer survivors found that greater adherence to the Mediterranean–DASH Intervention for Neurodegenerative Delay (MIND) diet, which emphasizes antioxidant-rich foods, was associated with better cognitive performance [15]. To assess the overall oxidative potential of dietary intake, the dietary oxidative balance score (DOBS) integrates dietary antioxidant and pro-oxidant exposures, with higher scores being associated with better cognitive function among older adults [16]. Our previous cross-sectional study found that higher DOBS was associated with lower odds of CRCI among patients with lung cancer [17]. Nevertheless, existing evidence remains largely observational, and robust interventional studies investigating whether antioxidant-focused dietary intervention can improve CRCI are still lacking.

Previous randomized controlled trials (RCTs) have suggested that antioxidant-related supplements or healthy dietary patterns may improve cognitive outcomes in populations not specifically defined by CRCI, including healthy adults, older adults with mild cognitive impairment (MCI), and patients with metastatic breast cancer [18,19,20,21]. However, these studies mainly evaluated nutraceutical supplements, isolated nutrient supplementation, or general healthy dietary patterns, rather than a structured antioxidant-focused dietary intervention incorporating dietary oxidative balance. Whether such an intervention can benefit patients with established CRCI, particularly those with lung cancer, remains unclear. Therefore, we conducted a 12-week assessor-blinded RCT to determine whether an antioxidant-focused dietary intervention could improve cognitive function, oxidative stress status, and QoL in lung cancer patients with established CRCI. We hypothesized that the intervention would improve cognitive outcomes while also promoting favorable changes in dietary oxidative balance, oxidative stress status, and QoL.

## 2. Materials and Methods

### 2.1. Study Design and Participants

This assessor-blinded, two-arm, parallel-group RCT was conducted at the Affiliated Hospital of Jiangnan University and was reported in accordance with the CONSORT 2025 statement. Participants’ recruitment was performed between 13 February and 30 September 2025 following the inclusion criteria: aged ≥18 years; histopathologically confirmed lung cancer without brain metastasis; meeting the operational definition of CRCI based on the Montreal Cognitive Assessment (MoCA); scheduled to receive anticancer treatment with an expected treatment duration of ≥3 months; and having a regular caregiver. Written informed consent was obtained from all participants before enrollment in the study. Exclusion criteria were: the presence of other malignancies; neurological or psychiatric disorders that could affect cognitive function; history of head trauma, cerebrovascular diseases or neurodegenerative disorders; current use of psychiatric medications or antioxidant-related dietary supplements; and incomplete clinical data or questionnaires.

### 2.2. Sample Size Calculation

The sample size calculation was based on the MoCA total score, which was the primary outcome of the study. The required sample size was estimated based on the following formula:

$$n=(Z_{1-\alpha /2}+Z_{1-\beta }{)}^{2}\times 2\sigma^{2}/\delta^{2}$$

The parameters used for the sample size calculation were derived from preliminary data of 20 participants in the pilot phase of the same study, with 10 participants in each group. These participants were not included in the formal RCT. At week 12, the mean MoCA scores were 23.20 ± 4.10 in the intervention group and 19.90 ± 6.08 in the control group. Accordingly, the expected between-group difference in week-12 MoCA scores (*δ*) was 3.30 points, and the pooled standard deviation (*σ*) was 5.19. The calculation assumed a two-sided significance level (*α*) of 0.05 and 80% power, yielding 39 participants per group. Allowing for a potential 20% loss to follow-up, the required sample size increased to 49 participants per group. Ultimately, 100 eligible participants were enrolled, with 50 individuals in each group.

### 2.3. Randomization and Masking

Following the completion of baseline assessment, participants were randomly allocated (1:1) to either the intervention group or the control group using a block randomization scheme with a fixed block size of four. An independent research assistant, who had no role in participant recruitment, intervention delivery, or outcome assessment, generated the random allocation sequence using R software (version 4.4.2). Allocation concealment was ensured through sequentially numbered, opaque, sealed envelopes prepared by the independent research assistant. The envelopes were opened sequentially only after completion of baseline assessments by designated research staff not involved in outcome assessment or statistical analysis.

Because of the behavioral nature of the dietary intervention, blinding of participants and intervention providers could not be implemented. Nevertheless, outcome assessors, laboratory technicians, and data analysts remained blinded throughout the study. To minimize contamination between groups, interventions were delivered separately, and educational materials were distributed only to participants in their assigned groups.

### 2.4. Intervention

Participants who were allocated to the control group received standard dietary counseling based on the Dietary Guidelines for Chinese Residents and the Chinese Dietary Pagoda. Counseling was delivered during hospitalization through a single face-to-face session. During follow-up, participants could additionally receive routine health guidance and consultation via WeChat or telephone as needed.

In addition to standard dietary counseling, participants in the intervention group received a 12-week antioxidant-focused dietary intervention program (Table 1). The intervention was delivered by trained nutrition educators through a combination of face-to-face sessions and structured online support via WeChat or telephone. To ensure intervention fidelity, all nutrition educators completed standardized training and implemented the intervention according to a structured protocol. The intervention process was supervised by senior researchers throughout the study to ensure consistency across educators.

The program focused on promoting increased consumption of antioxidant-rich foods and improving dietary oxidative balance through individualized dietary guidance, behavioral reinforcement, dietary self-monitoring, and adherence support. The quantitative dietary targets were introduced during the first in-hospital session and reinforced throughout the subsequent phases. Key targets included vegetables 300–500 g/day, with ≥50% being dark-colored vegetables; fruits 200–350 g/day, with an emphasis on dark-colored varieties; whole grains 75–150 g/day; nuts 15–20 g/day; legumes 25 g/day; and fish 300–500 g/week, consumed at least twice weekly. Additional targets for plant oils and meat, foods to be limited, examples of antioxidant-rich foods, recommended cooking methods, and dietary self-monitoring procedures are provided in Supplementary Table S1. During follow-up, nutrition educators reviewed participants’ dietary records, answered diet-related questions, and provided individualized feedback according to dietary intake. Participants were also advised to increase their intake of antioxidant-rich foods as part of their daily diets and were provided with practical recommendations regarding food selection and preparation methods to facilitate long-term adherence.

Intervention adherence was monitored throughout the home-based phases according to participants’ engagement with two core intervention activities: submission of dietary records and interaction with nutrition educators. During each phase, no dietary record submission was assigned 0 points, one submission was assigned 1 point, and two or more submissions were assigned 2 points. Similarly, no interaction with a nutrition educator was assigned 0 points, one interaction was assigned 1 point, and two or more interactions were assigned 2 points. The resulting adherence score ranged from 0 to 4 points per phase and from 0 to 16 points across the four home-based phases. To facilitate interpretation, each participant’s total score was converted to a percentage using the following formula: adherence percentage = (observed total score/16) × 100%. For descriptive purposes, adherence was operationally categorized as high (≥80%), moderate (50% to <80%), low (>0% to <50%), or no recorded adherence activity (0%). Because the total adherence score could take only integer values from 0 to 16, these thresholds corresponded to scores of 13–16 for high, 8–12 for moderate, 1–7 for low, and 0 for no recorded adherence activity. These study-specific thresholds were used solely for descriptive classification and were not intended to represent externally validated adherence cutoffs. The adherence analysis included all 50 participants initially allocated to the intervention group. A cumulative adherence score reflected participants’ engagement with the prescribed intervention activities and was not interpreted as evidence of achievement of dietary targets or clinical response. A phase-specific score greater than 0 indicated completion of at least one prescribed adherence activity during that phase. For participants who discontinued the intervention, adherence scores observed before discontinuation were retained, whereas both components in each subsequent phase were assigned 0 points because no further dietary records were submitted and no further interactions with nutrition educators occurred. For participants with a score of 0 during any phase, the research team proactively contacted them to identify barriers to engagement and addressed these issues during subsequent intervention sessions. The phase-specific engagement rate was calculated as the number of participants with an adherence score greater than 0 in each phase divided by all participants randomized to the intervention group (*n* = 50).

### 2.5. Baseline Characteristics

Baseline characteristics included sociodemographic variables (age, years of education, family monthly income, and marital status), clinical characteristics (treatment modality, cancer stage, surgical history, and comorbidities), and anthropometric measurements. Participants’ height and weight were obtained by trained staff according to standardized procedures in the morning after an overnight fast.

### 2.6. Outcomes

The primary outcome was the change in cognitive function assessed by the MoCA score from baseline to week 12. Secondary outcomes included CRCI status, DOBS, and QoL. Changes in oxidative stress biomarkers were considered exploratory mechanistic outcomes to investigate potential biological pathways underlying the intervention effects.

#### 2.6.1. Cognitive Function

Cognitive function was assessed at baseline and week 12 using the MoCA, a standardized, examiner-administered, performance-based screening tool for CRCI. Assessments were conducted by trained outcome assessors who were not involved in intervention delivery and remained blinded to group allocation. At both time points, the MoCA was administered in a quiet and private setting using standardized instructions and prespecified scoring procedures. Assessors used only the prompts permitted by the MoCA administration protocol and did not provide coaching, discuss participants’ dietary adherence or intervention experiences, or give evaluative feedback during the assessment. These procedures were intended to minimize assessment and expectancy biases. The MoCA comprises seven domains: visual and executive function, naming, attention, language, abstraction, memory and delayed recall, and orientation. Total MoCA scores range from 0 to 30, with higher scores reflecting better cognitive function. For study eligibility, cognitive impairment was operationally identified using education-adjusted MoCA cutoffs: ≤13 for participants without formal education, ≤19 for those with 1–6 years of education, and ≤24 for those with 7 or more years of education [22].

#### 2.6.2. Oxidative Stress Biomarkers

**Figure 1:**
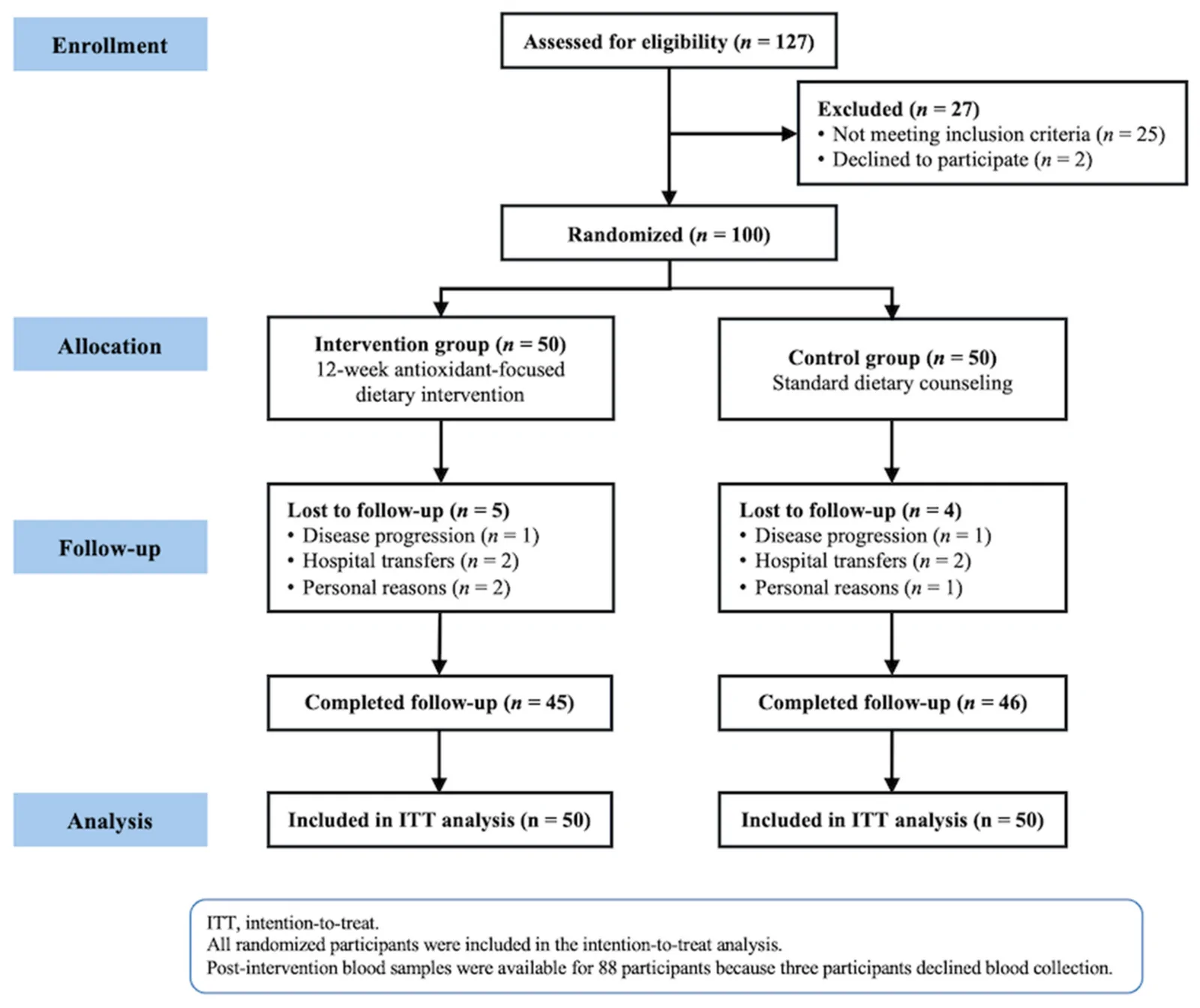
The flow diagram of participant recruitment in this trial.

Fasting venous blood samples were collected prior to scheduled anticancer treatment at baseline and after the 12-week intervention. Post-intervention blood samples were obtained from 88 participants (Figure 1). Following centrifugation at 3500 rpm for 10 min, plasma was isolated and stored until analysis. Oxidative stress biomarkers, including malondialdehyde (MDA), SOD, GPx, glutathione (GSH), and catalase (CAT), were determined using commercial assay kits (Nanjing Jiancheng Bioengineering Institute, Nanjing, China) according to the manufacturer’s instructions. All assays were performed in triplicate. Analytical reliability was evaluated using coefficients of variations (CVs) calculated from triplicate measurements of individual samples. Across all five oxidative stress biomarkers, most CVs for individual samples were below 10%, while only a small proportion ranged from 10% to 15%. No individual sample had a CV exceeding 15%.

#### 2.6.3. Dietary Intake Assessment and Dietary Oxidative Balance Score

Dietary intake was assessed at baseline and week 12 using three non-consecutive 24 h dietary recalls. These protocol-defined dietary assessments were used for nutrient intake estimation and calculation of the DOBS. The recalls were administered by trained research assistants who were not involved in delivering the dietary intervention. Before each recall, participants received standardized, non-judgmental instructions emphasizing that the purpose was to document their actual dietary intake rather than to evaluate their adherence to the dietary recommendations. They were informed that there were no right or wrong answers, that deviations from the recommendations were common and equally important to the study, and that their responses would not affect their clinical care or continued participation in the study. Research assistants used neutral, non-leading prompts and avoided providing evaluative feedback during the recalls. To improve recall accuracy, standardized food models and portion-size atlases were used during interviews. At each assessment time point, trained research staff actively contacted participants and scheduled the three recalls. Missed recalls were followed up and, whenever possible, rescheduled within the predefined assessment window. One dietary recall was conducted during hospitalization, and two additional recalls (one weekday and one weekend day) were completed after discharge via WeChat to better capture habitual dietary intake.

Considering that treatment-induced gastrointestinal symptoms may transiently affect food intake, dietary assessments after discharge were conducted only after symptom stabilization. Participants or caregivers were instructed to weigh and photograph foods, cooking oils, and spices using kitchen scales before submitting records via WeChat version 8. Dietary records were subsequently reviewed and verified through video communication with trained research assistants. Daily nutrient intakes were estimated using the Nutrition Calculator software (version 2.8.3.0, Beijing, China), with nutrient values derived from the *China Food Composition Tables* [23]. This software version has been widely applied in previous Chinese dietary intake studies for nutrient estimation [24]. Average daily nutrient intake at baseline and week 12 was calculated from the three recalls obtained at each assessment time point. Dietary records collected during the four home-based intervention phases were used solely for intervention delivery and adherence monitoring and were not included in nutrient intake estimation or DOBS calculation.

The DOBS was derived from the dietary components of the oxidative balance score (OBS) developed by Cho et al. [25]. It comprised 12 dietary components: three classified as pro-oxidants and nine as antioxidants. The pro-oxidant components were saturated fatty acids (SFAs), n-6 polyunsaturated fatty acids (n-6 PUFAs), and iron, whereas the antioxidant components were n-3 polyunsaturated fatty acids (n-3 PUFAs), monounsaturated fatty acids (MUFAs), dietary fiber, vitamins C, E, A, β-carotene, selenium, and zinc. At each assessment, dietary data from the intervention and control groups were pooled to derive component-specific tertile cutoffs among participants with available dietary data. For pro-oxidant components, scores of 2, 1, and 0 were assigned to participants in the lowest, middle, and highest tertiles, respectively. The scoring scheme was applied in the opposite direction for antioxidant components. The overall DOBS was calculated by summing the scores assigned to all components. Greater scores indicated a more favorable dietary oxidative balance.

#### 2.6.4. Quality of Life

QoL was assessed using the 12-Item Short-Form Health Survey (SF-12, version 2). The Chinese SF-12 has demonstrated acceptable reliability and validity among community-dwelling older adults [26]. The instrument comprises 12 items representing eight health dimensions: physical functioning, role physical, bodily pain, general health, vitality, social functioning, role emotional, and mental health [27]. Scores were summarized as the Physical Component Summary (PCS) and Mental Component Summary (MCS) according to the standard SF-12 version 2 scoring algorithm. Higher scores indicate better health-related QoL.

### 2.7. Statistical Analyses

All statistical procedures were conducted using R software (version 4.5.0). The distribution of continuous variables was assessed with the Shapiro–Wilk test. Normally distributed variables were reported as the mean ± standard deviation (SD), whereas skewed variables were expressed as median with the first and third quartiles (Q1, Q3). Baseline characteristics were compared between groups using independent-samples t-tests or Mann–Whitney U tests for continuous variables and chi-square tests, continuity-corrected chi-square tests, or Fisher’s exact tests for categorical variables, as appropriate.

For the categorical analysis of CRCI status at week 12, analyses were restricted to participants with available week-12 MoCA assessments. The proportions of participants meeting the CRCI criteria were compared between groups using Pearson’s chi-squared test. The unadjusted risk ratio (RR) and its 95% confidence interval (CI) were calculated from the corresponding 2 × 2 contingency table using the standard logarithmic (Katz) method, with the control group serving as the reference. No covariate adjustment or missing-data imputation was applied to this categorical analysis.

An intention-to-treat (ITT) approach was adopted for all efficacy analyses on continuous outcomes, in which all randomized participants were retained. Intervention effects over time were estimated using linear mixed-effects models (LMMs). This approach accommodates incomplete follow-up data under the missing-at-random (MAR) assumption. The LMMs included Group, Time, and Group × Time interaction as fixed-effect terms, with the control group and baseline assessment serving as reference categories. Within this parameterization, the coefficient for Group estimates the baseline difference between study arms, the coefficient for Time reflects temporal change in the control group, and the Group × Time interaction quantifies the intervention effect. A random intercept for each participant was incorporated to account for within-subject correlations arising from repeated measurements. Covariates included in the adjusted models were selected based on clinical relevance and previously reported associations with cognitive outcomes, including age, sex, years of education, cancer stage, and treatment modality [28,29]. Because BMI showed a borderline between-group difference at baseline, a sensitivity analysis was conducted by additionally adjusting for baseline BMI. Multicollinearity was evaluated using variance inflation factors (VIFs), with all variables showing VIFs < 5, indicating no evidence of problematic multicollinearity. As oxidative stress biomarkers generally exhibit right-skewed distributions, log transformation was performed prior to analysis. Intervention effects are reported as regression coefficients (*β*) with corresponding 95% CIs. Effect sizes were estimated using Cohen’s *d*, calculated as the between-group difference in estimated marginal means divided by the model-based residual standard deviation. Cohen’s *d* values of 0.2, 0.5, and 0.8 were interpreted as small, medium, and large effect sizes, respectively [30]. Given that CRCI classification was based on education-adjusted MoCA thresholds, subgroup analyses stratified by years of education were performed to explore potential effect modification. LMMs were conducted using the R packages lme4 version 4.5.0, lmerTest, and emmeans. A two-sided *p* < 0.05 was considered statistically significant. No formal adjustment for multiple comparisons was applied because MoCA was prespecified as the primary outcome, whereas secondary outcomes and biomarker analyses were considered supportive or exploratory analyses.

## 3. Results

### 3.1. Overview

Following eligibility assessment of 127 patients, 100 were enrolled and randomized to either the intervention group or the control group (*n* = 50 each). During the 12-week study period, nine participants withdrew from the study because of disease progression, hospital transfer, or personal reasons. Consequently, 45 participants (90.0%) in the intervention group and 46 participants (92.0%) in the control group completed the study. Among all 50 participants initially allocated to the intervention group, cumulative adherence scores across the four home-based phases were classified as high in 29 participants (58.0%), moderate in 13 (26.0%), and low in 8 (16.0%). No participants were classified as having no recorded adherence activity because all participants completed at least one prescribed adherence activity during the first home-based phase. The phase-specific engagement rates were 100.0%, 98.0%, 94.0%, and 90.0% across phases 1–4, respectively. No statistically significant differences in demographic, clinical, or treatment-related baseline characteristics were observed between patients who withdrew and those who completed the trial, with *p* values ranging from 0.060 to 1.000 (Supplementary Table S2). The trial was completed as planned after the target sample size had been reached and all scheduled 12-week intervention and follow-up assessments had been completed. No early termination occurred. No intervention-related adverse events were observed. Participant enrollment, randomization, follow-up, and analysis are illustrated in Figure 1.

### 3.2. Participant Baseline Characteristics

Baseline demographic, clinical, and lifestyle characteristics are shown in Table 2. The study population had a mean age of 66.10 ± 5.39 years, and most participants were male (78.0%). Most participants were diagnosed with non-small cell lung cancer, and 70.0% had stage III–IV disease. The distribution of anticancer treatment modalities was comparable between groups (*p* > 0.05). Likewise, no significant between-group differences were observed for any other baseline demographic, clinical, and lifestyle variables (all *p* > 0.05).

### 3.3. Cognitive Outcomes

#### 3.3.1. Change in MoCA Scores

The mean MoCA score increased from 17.28 ± 3.59 at baseline to 19.44 ± 4.87 at week 12 in the intervention group, whereas it remained relatively stable in the control group, changing from 17.86 ± 4.19 to 17.65 ± 3.96.

As shown in Table 3, neither the main effect of Group nor the main effect of Time was statistically significant, whereas the Group × Time interaction term reached statistical significance across all analytical models, with the intervention group demonstrating a greater increase in MoCA scores than the control group. In the fully adjusted model (Model 3), the adjusted between-group difference in mean change was 2.626 points (95% CI: 0.825, 4.427; *p* = 0.005), corresponding to a large effect size (Cohen’s *d* = 0.84). The results of the sensitivity analysis, which additionally adjusted for baseline BMI, were consistent with those of the primary adjusted model (Supplementary Table S3), supporting the robustness of the findings.

#### 3.3.2. Change in the Proportion of CRCI

**Figure 2:**
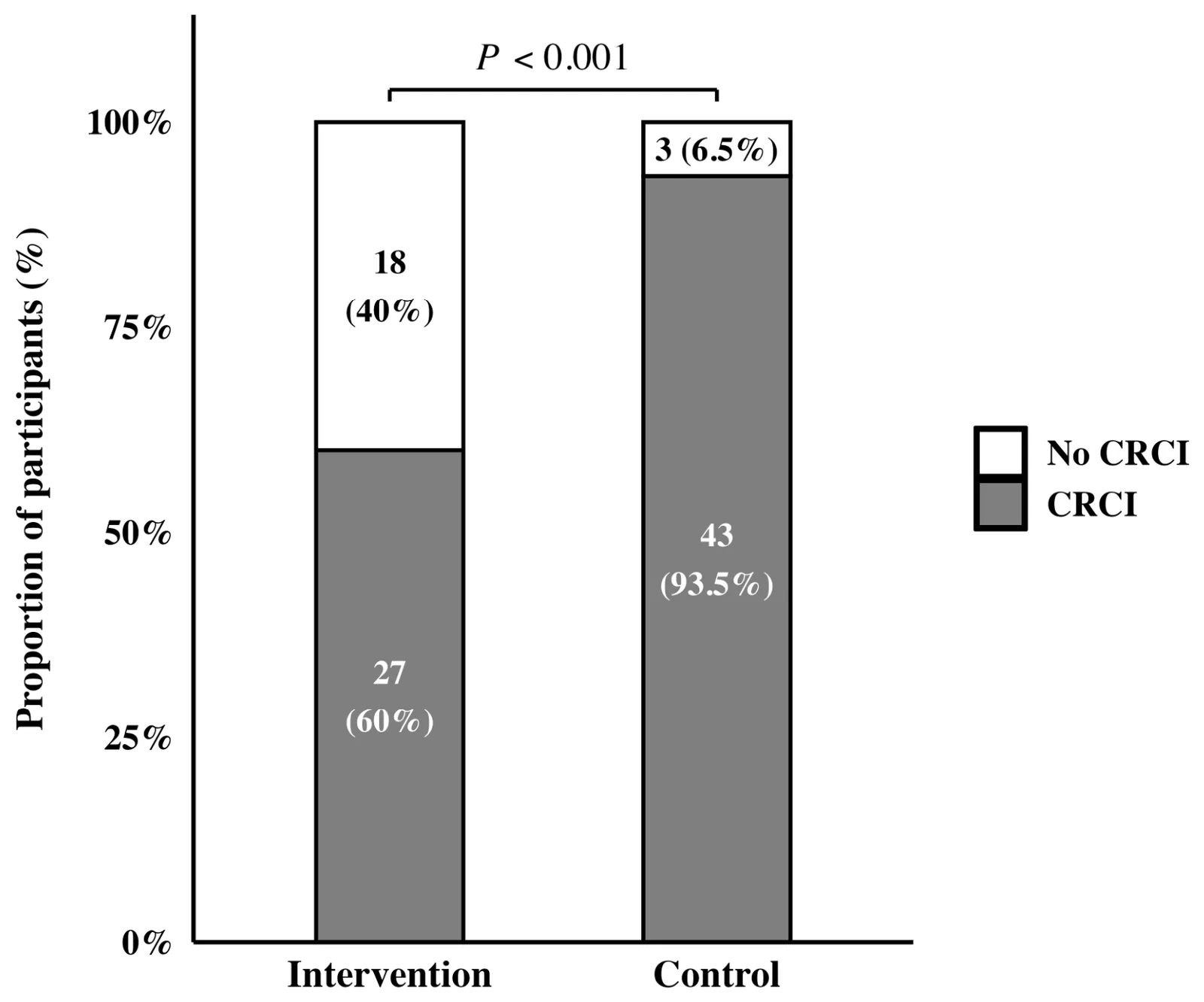
CRCI status at week 12 among participants with available week-12 data. The *p*-value was derived from a between-group chi-squared test comparing the proportion of participants meeting CRCI criteria at week 12. CRCI, cancer-related cognitive impairment.

In the complete-case analysis (*n* = 91), the proportion of participants meeting CRCI criteria at week 12 was markedly lower in the intervention group than in the control group (60.0% vs. 93.5%, *p* < 0.001; Figure 2). The corresponding unadjusted RR was 0.64 (95% CI: 0.50, 0.82), indicating a reduced risk of CRCI classification in the intervention group.

#### 3.3.3. Exploratory Subgroup Analysis by Educational Attainment

In exploratory subgroup analyses stratified by years of education, the estimated Group × Time interaction coefficients were positive and statistically significant across all educational subgroups (all *p* < 0.05; Table 4).

### 3.4. Effects on DOBS

Over 12 weeks, the mean DOBS increased from 12.44 ± 2.98 to 13.60 ± 2.52 in the intervention group but decreased from 12.24 ± 3.30 to 11.09 ± 2.61 in the control group. Descriptive data showed increased intakes of MUFAs, n-3 PUFAs, β-carotene, vitamins C and E, and dietary fiber and decreased SFAs intake in the intervention group. Conversely, the control group showed decreased MUFAs and vitamin C intakes and increased SFAs and n-6 PUFAs intakes (Supplementary Table S4).

As presented in Table 5, no significant main effects of Group or Time were detected, whereas the Group × Time interaction remained significant across all three models, indicating greater improvements in dietary oxidative balance in the intervention group relative to the control group. Model 3 estimated a 2.292-point difference in mean change between the groups (*p* = 0.006), corresponding to a large effect size (Cohen’s *d* = 0.80). The results of the sensitivity analysis, which additionally adjusted for BMI, were consistent with those of the primary adjusted model (Supplementary Table S3), supporting the robustness of the findings.

### 3.5. Effects on Oxidative Stress Biomarkers

**Figure 3:**
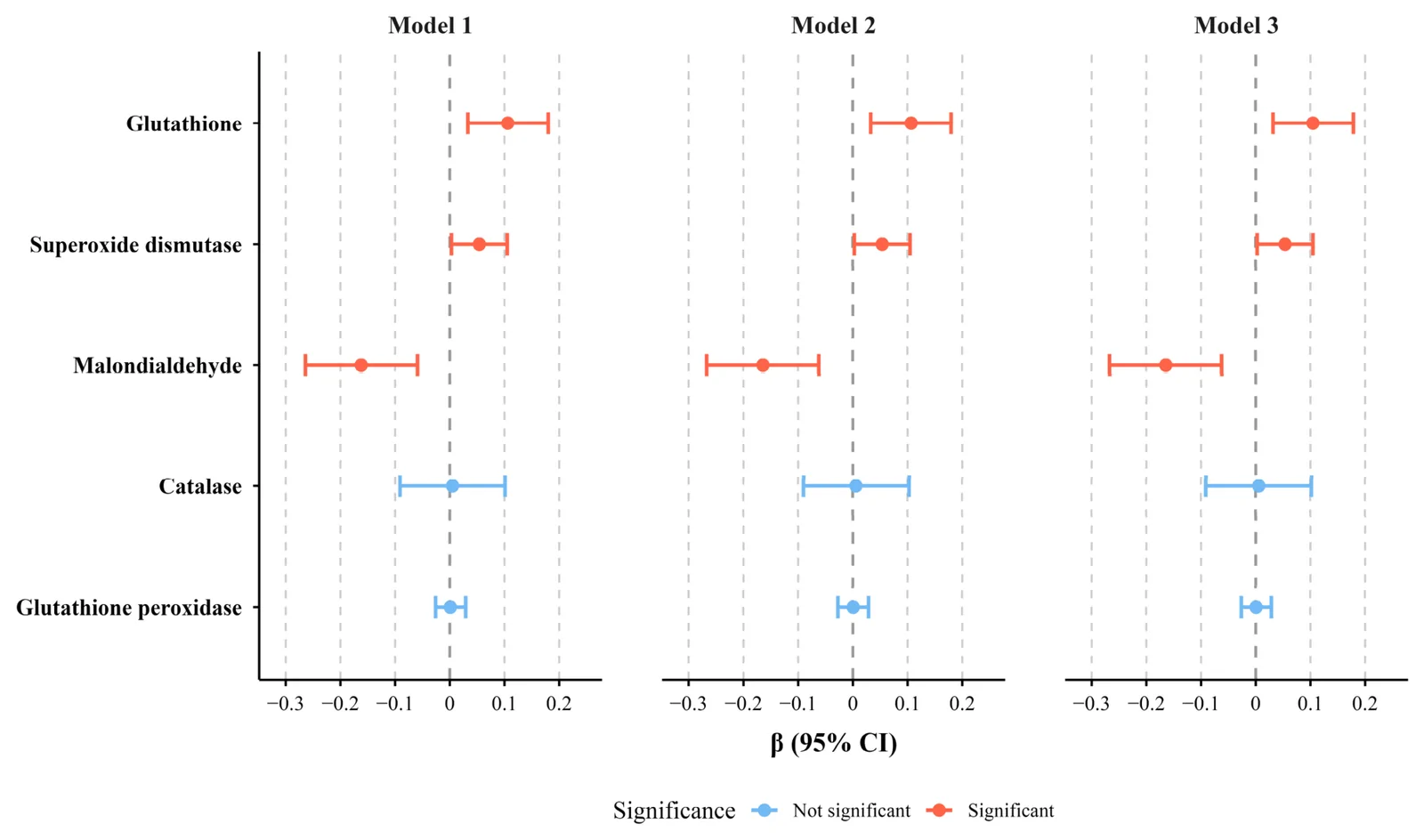
Group × Time effects of the antioxidant-focused dietary intervention on oxidative stress biomarkers. The plot presents the regression coefficients (*β*) and 95% confidence intervals (CIs) for the Group × Time interaction term derived from linear mixed-effects models, with all estimates reported on the log-transformed scale. Positive coefficients indicate greater increases in biomarker levels in the intervention group relative to the control group, whereas negative coefficients indicate greater reductions. Model 1: unadjusted. Model 2: adjusted for age, sex, and years of education. Model 3: adjusted for age, sex, years of education, cancer stage, and treatment modality.

Descriptive statistics of oxidative stress biomarkers at baseline and week 12 are summarized in Supplementary Table S5. In the fully adjusted model (Model 3, Figure 3), significant Group × Time effects were observed for GSH (*β* = 0.105; 95% CI: 0.032, 0.179; *p* = 0.006; Cohen’s *d* = 0.84), SOD (*β* = 0.054; 95% CI: 0.004, 0.105; *p* = 0.036; Cohen’s *d* = 0.63), and the lipid peroxidation marker MDA (*β* = −0.164; 95% CI: −0.267, −0.062; *p* = 0.002; Cohen’s *d* = 0.94). No significant Group × Time interactions were observed for CAT (*β* = 0.006; 95% CI: −0.090, 0.103; *p* = 0.897) or GPx (*β* = 0.001; 95% CI: −0.027, 0.029; *p* = 0.931) in Model 3. The estimates showed consistent directions across Models 1–3 and were not materially altered by additional adjustment for baseline BMI (Supplementary Table S3).

### 3.6. Effects on QoL

**Figure 4:**
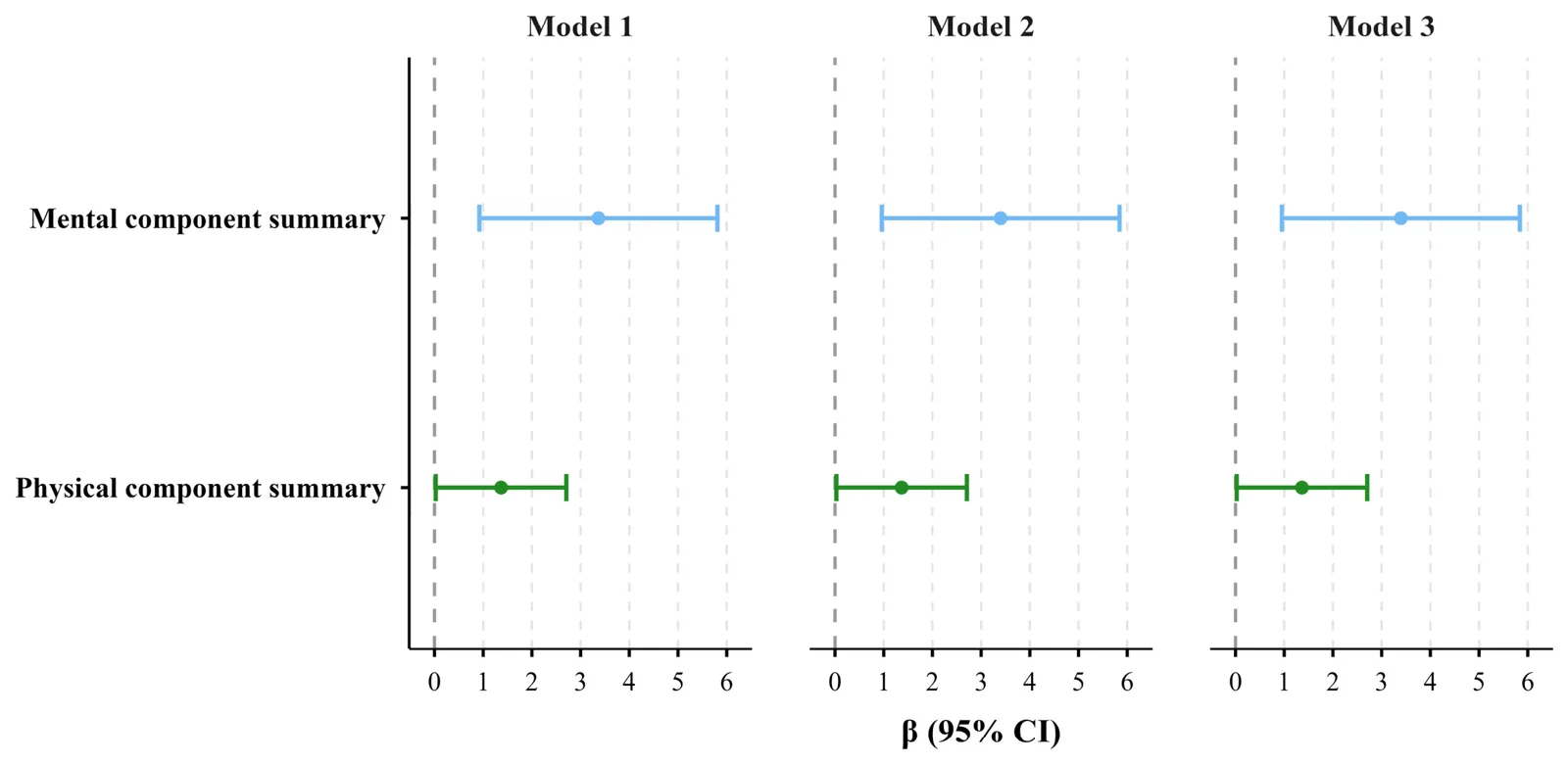
Effects of the antioxidant-focused dietary intervention on QoL outcomes. The plot presents the regression coefficients (*β*) and 95% confidence intervals (CIs) for the Group × Time interaction term derived from linear mixed-effects models. Positive coefficients indicate greater improvements in quality-of-life scores over time in the intervention group compared with the control group. Model 1: unadjusted. Model 2: adjusted for age, sex, and years of education. Model 3: adjusted for age, sex, years of education, cancer stage, and treatment modality.

Descriptive statistics for PCS and MCS at baseline and week 12 are presented in Supplementary Table S6. In Model 3 (Figure 4), significant Group × Time effects were observed for PCS (*β* = 1.364; 95% CI: 0.024, 2.704; *p* = 0.046; Cohen’s *d* = 0.60) and MCS (*β* = 3.395; 95% CI: 0.952, 5.838; *p* = 0.007; Cohen’s *d* = 0.81). Sensitivity analyses with additional adjustment for baseline BMI did not materially change the results for either outcome (Supplementary Table S3).

## 4. Discussion

The present RCT demonstrated that an antioxidant-focused dietary intervention for 12 weeks improved cognitive function and QoL in lung cancer patients with CRCI. These benefits were accompanied by improvements in dietary oxidative balance and oxidative stress biomarkers, supporting a potential role of oxidative stress modulation in the observed cognitive improvements. Given the limited effectiveness of current pharmacological options for CRCI, the antioxidant-focused dietary intervention may provide a practical supportive-care strategy that could be incorporated into the management of lung cancer patients with CRCI.

In recent years, dietary interventions have attracted increasing attention as potential strategies in the management of cognitive impairment. In the present study, the adjusted between-group difference in MoCA change was 2.63 points, corresponding to a large standardized effect (Cohen’s *d* = 0.84), suggesting that the 12-week antioxidant-focused dietary intervention significantly improved cognitive function among lung cancer patients with CRCI. This finding is consistent with previous studies demonstrating cognitive benefits associated with antioxidant-rich dietary patterns. For example, an RCT using fruit- and vegetable-based nutritional supplementation reported improvements across several aspects of cognition, including short-term memory, working memory, selective and sustained attention, and processing speed [18]. Similarly, supplementation with high-dose n-3 PUFAs and n-6 PUFAs together with antioxidant vitamins was associated with slower deterioration in cognitive and functional outcomes among older adults with MCI [19]. Moreover, the PREDIMED trial demonstrated that adherence to a Mediterranean dietary pattern supplemented with extra-virgin olive oil or nuts significantly enhanced cognitive performance compared with a low-fat diet [20]. However, no validated minimal clinically important difference has been established for MoCA changes in patients with CRCI; therefore, whether the observed intervention-related improvement represents a clinically meaningful change remains uncertain. Direct quantitative comparison with previous nutritional intervention studies is also difficult because of differences in study populations, intervention designs, and cognitive outcome measures. Therefore, although the magnitude observed in the present study is encouraging, whether it translates into clinically meaningful functional benefits requires confirmation using comprehensive neuropsychological assessments and patient-reported or functional outcomes.

Importantly, the cognitive benefits observed in the present study were achieved through modification of the overall dietary pattern rather than supplementation with isolated nutrients. Evidence regarding isolated nutrient supplements remains inconsistent. For instance, supplementation with n-3 PUFAs (fish oil) failed to significantly enhance cognitive performance in cognitively healthy older adults [31], while a large-scale trial involving 7540 older men found no significant benefits on the onset of dementia following vitamin E or selenium supplementation [32]. By contrast, a randomized, placebo-controlled trial showed that docosahexaenoic acid supplementation had beneficial effects on cognitive function and slowed the progression of hippocampal atrophy among older adults with MCI [33]. These inconsistencies may reflect differences in study populations, baseline cognitive status, intervention duration, and intervention composition. Collectively, these findings indicate that cognitive impairment is a multifactorial condition and that dietary patterns may offer broader cognitive benefits by providing a range of antioxidant and bioactive compounds capable of acting synergistically across multiple biological pathways.

Descriptive dietary data indicated increases in several antioxidant dietary components and a reduction in SFA intake in the intervention group. Together with the moderate or high adherence scores and sustained engagement with the intervention activities observed among most participants, these findings suggest that at least some of the recommended dietary behaviors were implemented. Such partial implementation of these dietary recommendations may have contributed to the observed intervention effects, whereas lower engagement among some participants may have attenuated these effects. However, the study-specific adherence score reflected participants’ engagement with intervention activities (dietary record submissions and interactions with nutrition educators) rather than direct achievement of the prescribed dietary targets. As no formal adherence–outcome or dose–response analysis was performed, the extent to which adherence affected the observed intervention effects remains uncertain. Notably, DOBS increased significantly following the intervention. DOBS reflects the balance between dietary antioxidants and pro-oxidants and provides an integrated measure of the oxidative potential of habitual dietary intake [34]. By integrating information from multiple dietary components, DOBS may better reflect real-world dietary exposure than approaches focusing on individual nutrients. The intervention-induced increase in DOBS not only suggests successful adoption of the recommended dietary pattern but also provides indirect evidence that the intervention achieved meaningful dietary modification, thereby strengthening the interpretation of the observed clinical outcomes. A previous observational study reported an inverse association between DOBS and CRCI among lung cancer patients [17]. Similar findings have also been reported in studies evaluating oxidative balance scores in general populations, where higher scores were associated with improved cognitive function and a reduced risk of cognitive decline [16,35,36]. While previous studies have largely been observational, the present trial extends the evidence by demonstrating that dietary oxidative balance can be modified through a structured dietary intervention and that such modification is accompanied by improvements in cognitive outcomes. Importantly, the concurrent improvements in DOBS, cognitive outcomes, and oxidative stress biomarkers are consistent with, but do not establish, the possibility that oxidative stress processes may be involved in the association between dietary oxidative balance and cognition.

To explore potential biological processes associated with the observed cognitive benefits, we examined changes in oxidative stress biomarkers, given accumulating evidence implicating oxidative stress in CRCI. Excessive production of ROS and impairment of endogenous antioxidant defenses may contribute to neuronal damage, synaptic dysfunction, and cognitive decline [37]. Cancer progression and its treatment may further exacerbate oxidative stress by increasing ROS generation and disrupting redox homeostasis, thereby promoting oxidative damage to lipids, proteins, and nucleic acids [38]. Consequently, persistent oxidative stress has been recognized as an important contributor to cognitive decline in cancer patients [39]. In our study, the antioxidant-focused dietary intervention significantly increased GSH and SOD levels while reducing MDA concentrations, indicating enhanced endogenous antioxidant defenses and decreased lipid peroxidation. These favorable changes have also been reported in previous dietary intervention studies. For instance, an RCT demonstrated that a polyphenol-rich diet significantly reduced urinary 8-isoprostanes, indicating attenuation of lipid peroxidation [40]. Similarly, another RCT showed that a high-antioxidant diet increased SOD and GPx activities while reducing MDA and lipid hydroperoxide levels [41]. Evidence from DASH interventions has also demonstrated increases in GSH and reductions in MDA concentrations, together with favorable trends in other oxidative stress biomarkers [42]. Nevertheless, not all intervention studies have reported consistent findings. A crossover trial involving healthy participants found that daily consumption of walnuts for six weeks did not significantly alter MDA levels or total antioxidant capacity [43]. Likewise, short-term interventions aimed at increasing vegetable consumption have failed to produce significant changes in oxidative stress biomarkers [44]. Several factors may explain these discrepancies. First, intervention duration may be critical, as short-term dietary modifications may be insufficient to induce sustained biological adaptations. Second, baseline oxidative stress levels differ substantially across study populations. Individuals experiencing elevated oxidative stress may derive greater benefits from antioxidant-focused dietary interventions than healthy individuals with relatively balanced redox status. Third, responsiveness to antioxidant interventions may depend on the underlying oxidative stress burden. Patients with cancer may experience elevated oxidative stress associated with disease progression and anticancer treatment [45,46]. This heightened oxidative stress burden may partly explain why dietary modulation could produce more detectable changes in this population than in healthy individuals, although this possibility requires validation in future research. Notably, CAT and GPx did not change significantly in the present study. These biomarkers represent distinct but interconnected aspects of redox homeostasis. SOD catalyzes the conversion of superoxide radicals into hydrogen peroxide, which can subsequently be decomposed by CAT or reduced by GPx using GSH as a reducing substrate [47]. GPx also contributes to the removal of organic hydroperoxides, whereas MDA is a downstream product of lipid peroxidation and therefore reflects oxidative damage rather than antioxidant enzyme activity [47]. Accordingly, increases in SOD and GSH and a reduction in MDA may occur without detectable changes in CAT or GPx because these biomarkers represent different regulatory components within the redox network. The absence of statistically significant changes in CAT and GPx may reflect differential responses among individual components of this network, although the underlying mechanisms were not directly investigated in the present study. Collectively, these findings support oxidative stress modulation as a plausible biological mechanism through which an antioxidant-focused dietary intervention may contribute to cognitive improvements. Specifically, dietary antioxidants may enhance endogenous antioxidant defenses and attenuate lipid peroxidation. These effects could, in turn, help limit oxidative neuronal damage and synaptic dysfunction implicated in CRCI. However, because no formal mediation analysis was performed, the concurrent changes in these measures cannot establish that oxidative stress modulation mediated the cognitive benefits observed after the intervention.

Moreover, the directional concordance between DOBS and oxidative stress biomarkers should be interpreted cautiously. DOBS was calculated from 24 h dietary recalls, which rely on self-report and are susceptible to recall and social desirability bias. Participants in the intervention group received intensive dietary intervention focused on the recommended foods, which may have increased the likelihood of reporting dietary behaviors consistent with the intervention recommendations. Although oxidative stress biomarkers provide objective information on peripheral redox status, they are not specific indicators of dietary intake or intervention adherence and therefore cannot validate the self-reported dietary data used to derive DOBS. The concordance between these measures provides supportive evidence for biological plausibility, but potential reporting bias in DOBS remains an important limitation when interpreting the relationship between behavioral dietary measures and biochemical outcomes.

Given that anticancer treatment modality may influence both cognitive function and oxidative stress [11,12], its potential confounding effect was considered. Further adjustment for cancer stage and treatment modality did not materially alter the estimated intervention effects on MoCA scores or any of the five oxidative stress biomarkers, supporting the robustness of the findings to adjustment for these clinical factors. However, this study did not evaluate the specific effects of different treatment modalities on cognitive function or oxidative stress biomarkers. Therefore, the potential influence of specific agents, combination regimens, or cumulative treatment exposure cannot be excluded.

Beyond cognitive outcomes and oxidative stress, the antioxidant-focused dietary intervention was associated with significant improvements in both physical and mental dimensions of QoL. This finding is clinically relevant because CRCI affects not only cognitive performance but also broader aspects of daily functioning and psychological well-being. Previous studies have also reported improvements in QoL following dietary interventions among patients with cancer [48,49]. Several factors may help explain the observed benefits. Adoption of healthier dietary behaviors may be associated with better physical functioning and general health perceptions, while lower levels of oxidative stress may be related to fewer treatment-related symptoms that adversely affect daily life. In addition, cognitive impairment has been consistently associated with poorer QoL among cancer survivors [50]. In the present study, both cognitive function and QoL improved concurrently. This finding is consistent with previous evidence. A longitudinal study reported that executive function was associated with subsequent QoL [51]. In addition, an RCT reported improvements in QoL following cognitive training [52]. These findings suggest a possible association between cognitive function and QoL; however, because no mediation analysis was performed, the direction and underlying pathways of this relationship remain uncertain.

From a clinical perspective, an antioxidant-focused dietary intervention may represent a low-cost, safe, and potentially scalable strategy that could be readily incorporated into supportive care for patients with lung cancer. Unlike pharmacological approaches, dietary modification may simultaneously influence cognitive function, oxidative stress, and overall well-being. Although additional validation is warranted, these findings support further exploration of antioxidant-focused dietary counseling as a potential supportive-care strategy in oncology settings.

Several limitations should be acknowledged. First, given the behavioral nature of the dietary intervention, participant and provider blinding could not be achieved, raising the possibility of performance bias. Second, the intervention combined dietary counseling, behavioral support, caregiver involvement, and ongoing follow-up, making it difficult to determine the relative contribution of each component. Third, dietary intake was assessed using self-reported methods, which are susceptible to recall bias and social desirability bias. Fourth, although changes in oxidative stress biomarkers provide preliminary biological context for the findings, causal or mediating relationships between dietary modification, oxidative stress, and cognitive improvement cannot be definitively established. Fifth, cognitive function was assessed using the MoCA, a brief screening instrument rather than a comprehensive neuropsychological battery, which may have limited the detailed characterization of changes within individual cognitive domains. Additionally, the study was conducted at a single center, involved a relatively modest sample size, and included only lung cancer patients with CRCI, which may limit generalizability to other oncology populations and clinical settings. Finally, the 12-week follow-up period did not allow assessment of the long-term sustainability of the intervention effects. Therefore, larger multicenter studies involving more diverse oncology populations are warranted. Future studies should also formally evaluate the feasibility of implementing this intervention in routine oncology practice, incorporate comprehensive neuropsychological assessments and include longer follow-up periods.

## 5. Conclusions

In conclusion, this study demonstrated that an antioxidant-focused dietary intervention significantly improved cognitive function, dietary oxidative balance, oxidative stress status, and QoL among lung cancer patients with CRCI. The findings support the potential involvement of oxidative stress modulation in the relationship between dietary oxidative balance and cognitive outcomes. However, because the association between changes in oxidative stress biomarkers and cognitive outcomes was observational within this trial and no mediation analysis was performed, a causal mechanistic relationship cannot be established. Given its apparent short-term safety and feasibility, as well as its potential scalability, an antioxidant-focused dietary intervention may represent a promising supportive-care strategy for managing CRCI in patients with lung cancer. Future large-scale multicenter trials with longer follow-up durations are needed to determine the durability of the intervention effects and to further investigate the mechanisms contributing to these outcomes.

## Figures and Tables

**Table 1:** Structure of the antioxidant-focused dietary intervention.

Stage	Setting	Delivery Mode	Objective
Baseline	Pre-intervention	Face-to-face	Baseline assessment and rapport establishment
Stage 1 (Weeks 1–3)	In-hospital	Face-to-face; 30 min	Establish fundamental understanding of antioxidant nutrition and CRCI
	Post-discharge	Online (WeChat/telephone)	Initiate dietary behavior change
Stage 2 (Weeks 4–6)	In-hospital	Face-to-face; 30 min	Enhance dietary self-management skills
	Post-discharge	Online (WeChat/telephone)	Reinforce adherence to dietary behaviors
Stage 3 (Weeks 7–9)	In-hospital	Face-to-face; 30 min	Strengthen behavioral reinforcement and problem-solving
	Post-discharge	Online (WeChat/telephone)	Optimize dietary patterns and maintain engagement
Stage 4 (Weeks 10–12)	In-hospital	Face-to-face; 30 min	Consolidate long-term dietary behavior change
	Post-discharge	Online (WeChat/telephone)	Maintain long-term adherence and intervention effects

CRCI: Cancer-related cognitive impairment.

**Table 2:** Baseline characteristics of participants in the intervention and control groups.

Variable	Total (*n* = 100)	Intervention (*n* = 50)	Control (*n* = 50)	* t * /χ^2^	* p *
Age (years)	66.10 ± 5.39	65.86 ± 4.90	66.34 ± 5.88	0.443	0.658
BMI (kg/m^2^)	23.29 ± 2.97	23.80 ± 2.55	22.78 ± 3.29	−1.736	0.086
Sex					
Male	78 (78.0)	37 (74.0)	41 (82.0)	0.932	0.334
Female	22 (22.0)	13 (26.0)	9 (18.0)		
Smoking status					
Never	37 (37.0)	20 (40.0)	17 (34.0)	-	0.520
Former	57 (57.0)	26 (52.0)	31 (62.0)		
Current	6 (6.0)	4 (8.0)	2 (4.0)		
Alcohol use					
Never	46 (46.0)	24 (48.0)	22 (44.0)	-	0.762
Former	51 (51.0)	24 (48.0)	27 (54.0)		
Current	3 (3.0)	2 (4.0)	1 (2.0)		
Marital status					
Unmarried/divorced/widowed	2 (2.0)	0 (0.0)	2 (4.0)	0.510	0.475
Married	98 (98.0)	50 (100.0)	48 (96.0)		
Years of education					
No formal education	12 (12.0)	7 (14.0)	5 (10.0)	0.467	0.792
1–6 years	30 (30.0)	14 (28.0)	16 (32.0)		
≥7 years	58 (58.0)	29 (58.0)	29 (58.0)		
Employment					
Employed	1 (1.0)	0 (0.0)	1 (2.0)	-	0.436
Unemployed	6 (6.0)	2 (4.0)	4 (8.0)		
Retired	93 (93.0)	48 (96.0)	45 (90.0)		
Residence					
Rural areas	26 (26.0)	14 (28.0)	12 (24.0)	1.363	0.506
Towns	14 (14.0)	5 (10.0)	9 (18.0)		
Urban areas	60 (60.0)	31 (62.0)	29 (58.0)		
Family monthly income (CNY)					
<3000	15 (15.0)	7 (14.0)	8 (16.0)	1.490	0.475
3000–5000	39 (39.0)	17 (34.0)	22 (44.0)		
>5000	46 (46.0)	26 (52.0)	20 (40.0)		
Physical activity level					
Low	30 (30.0)	15 (30.0)	15 (30.0)	-	1.000
Moderate	63 (63.0)	32 (64.0)	31 (62.0)		
High	7 (7.0)	3 (6.0)	4 (8.0)		
Presence of comorbidities					
No	50 (50.0)	25 (50.0)	25 (50.0)	0.000	1.000
Yes	50 (50.0)	25 (50.0)	25 (50.0)		
Pathology subtype					
Non-small cell lung cancer	91 (91.0)	46 (92.0)	45 (90.0)	0.000	1.000
Small cell lung cancer	9 (9.0)	4 (8.0)	5 (10.0)		
Cancer stage					
I	18 (18.0)	9 (18.0)	9 (18.0)	0.521	0.914
II	12 (12.0)	6 (12.0)	6 (12.0)		
III	31 (31.0)	17 (34.0)	14 (28.0)		
IV	39 (39.0)	18 (36.0)	21 (42.0)		
Treatment modality					
Chemotherapy alone	20 (20.0)	11 (22.0)	9 (18.0)	1.021	0.796
Chemotherapy plus immunotherapy	26 (26.0)	12 (24.0)	14 (28.0)		
Targeted therapy-containing regimens	27 (27.0)	12 (24.0)	15 (30.0)		
Other systemic therapy	27 (27.0)	15 (30.0)	12 (24.0)		
History of surgery					
No	57 (57.0)	28 (56.0)	29 (58.0)	0.041	0.840
Yes	43 (43.0)	22 (44.0)	21 (42.0)		

Data are presented as *n* (%) or mean ± SD. Between-group comparisons were performed using the independent-samples t-test for continuous variables and Pearson’s chi-square test, the chi-square test with continuity correction, or Fisher’s exact test for categorical variables, as appropriate. CNY, Chinese yuan.

**Table 3:** Effects of the antioxidant-focused dietary intervention on MoCA scores.

Variable	Model 1		Model 2		Model 3		
	* β * (95% CI)	* p *	* β * (95% CI)	* p *	* β * (95% CI)	* p *	Cohen’s *d*
Group	−0.580 (−2.039, 0.879)	0.433	−0.362 (−1.603, 0.878)	0.564	−0.269 (−1.520, 0.982)	0.671	-
Time	−0.206 (−1.701, 1.289)	0.786	−0.325 (−1.589, 0.940)	0.612	−0.313 (−1.584, 0.958)	0.627	-
Group × Time	2.353 (0.239, 4.467)	0.029	2.619 (0.827, 4.410)	0.004	2.626 (0.825, 4.427)	0.005	0.84

Model 1: unadjusted. Model 2: adjusted for age, sex, and years of education. Model 3: adjusted for age, sex, years of education, cancer stage, and treatment modality.

**Table 4:** Exploratory subgroup analysis of intervention effects on MoCA scores stratified by educational attainment.

Variable	Sample Size		Model 1		Model 2		Model 3	
	Intervention	Control	* β * (95% CI)	* p *	* β * (95% CI)	* p *	* β * (95% CI)	* p *
No formal education	7	5	3.230 (0.126, 6.334)	0.043	3.158 (0.087, 6.229)	0.045	3.367 (0.241, 6.493)	0.037
1–6 years	14	16	3.016 (0.260, 5.771)	0.033	3.035 (0.274, 5.795)	0.032	3.029 (0.262, 5.795)	0.033
≥ 7 years	29	29	2.614 (1.064, 4.163)	0.001	2.611 (1.061, 4.161)	0.001	2.598 (1.041, 4.154)	0.002

Model 1: unadjusted. Model 2: adjusted for age and sex. Model 3: adjusted for age, sex, cancer stage, and treatment modality.

**Table 5:** Linear mixed-effects model analysis of changes in DOBS.

Variable	Model 1		Model 2		Model 3		
	* β * (95% CI)	* p *	* β * (95% CI)	* p *	* β * (95% CI)	* p *	Cohen’s *d*
Group	0.200 (−0.937, 1.337)	0.729	0.312 (−0.807, 1.431)	0.583	0.294 (−0.840, 1.429)	0.609	-
Time	−1.153 (−2.315, 0.009)	0.052	−1.142 (−2.282, 0.002)	0.051	−1.139 (−2.291, 0.014)	0.053	-
Group × Time	2.313 (0.665, 3.961)	0.006	2.281 (0.665, 3.898)	0.006	2.292 (0.656, 3.928)	0.006	0.80

Model 1: unadjusted. Model 2: adjusted for age, sex, and years of education. Model 3: adjusted for age, sex, years of education, cancer stage, and treatment modality.

## Data Availability

The data presented in this study are available on request from the corresponding author due to privacy and ethical restrictions related to the protection of participants’ personal health information.

## Supplementary Materials

The following supporting information can be downloaded at: https://www.mdpi.com/article/10.3390/antiox15080932/s1, Table S1. Structure and components of the antioxidant-focused dietary intervention. Table S2. Comparison of baseline characteristics between dropout and completed groups. Table S3. Sensitivity analyses of intervention effects after additional adjustment for baseline BMI. Table S4. Descriptive summary of DOBS and daily nutrient intakes at baseline and week 12. Table S5. Descriptive summary of oxidative stress biomarkers at baseline and at week 12. Table S6. Descriptive summary of quality of life at baseline and at week 12.

## Abbreviations

The following abbreviations are used in this manuscript:

BMI	Body mass index
CAT	Catalase
CI	Confidence interval
CNY	Chinese yuan
CRCI	Cancer-related cognitive impairment
CV	Coefficient of variation
DASH	Dietary Approaches to Stop Hypertension
DOBS	Dietary oxidative balance score
GPx	Glutathione peroxidase
GSH	Glutathione
ITT	Intention-to-treat
LMMs	Linear mixed-effects models
MAR	Missing-at-random
MCI	Mild cognitive impairment
MCS	Mental Component Summary
MDA	Malondialdehyde
MIND	Mediterranean–DASH Intervention for Neurodegenerative Delay
MoCA	Montreal Cognitive Assessment
MUFAs	Monounsaturated fatty acids
n-3 PUFAs	n-3 polyunsaturated fatty acids
n-6 PUFAs	n-6 polyunsaturated fatty acids
OBS	Oxidative balance score
PCS	Physical Component Summary
Q1	First quartile
Q3	Third quartile
QoL	Quality of life
RCT	Randomized controlled trial
ROS	Reactive oxygen species
RR	Risk ratio
SD	Standard deviation
SF-12	12-Item Short-Form Health Survey
SFAs	Saturated fatty acids
SOD	Superoxide dismutase
VIFs	Variance inflation factors

## References

[1] Siegel R.L., Miller K.D., Fuchs H.E., Jemal A. (2021). Cancer statistics, 2021. CA Cancer J. Clin..

[2] Ho M.H., So T.W., Fan C.L., Chung Y.T., Lin C.C. (2024). Prevalence and assessment tools of cancer-related cognitive impairment in lung cancer survivors: A systematic review and proportional meta-analysis. Support. Care Cancer.

[3] Bai L., Yu E. (2021). A narrative review of risk factors and interventions for cancer-related cognitive impairment. Ann. Transl. Med..

[4] Karschnia P., Parsons M.W., Dietrich J. (2019). Pharmacologic management of cognitive impairment induced by cancer therapy. Lancet Oncol..

[5] Zeng Y., Dong J., Huang M., Zhang J., Zhang X., Xie M., Wefel J.S. (2020). Nonpharmacological interventions for cancer-related cognitive impairment in adult cancer patients: A network meta-analysis. Int. J. Nurs. Stud..

[6] Seok J.W., Kim G., Kim J.U. (2024). Comparative efficacy of seven nonpharmacological interventions on global cognition in older adults with and without mild cognitive impairment: A network meta-analysis of randomized controlled trials. Sci. Rep..

[7] Gardener S.L., Rainey-Smith S.R., Barnes M.B., Sohrabi H.R., Weinborn M., Lim Y.Y., Harrington K., Taddei K., Gu Y., Rembach A. (2015). Dietary patterns and cognitive decline in an Australian study of ageing. Mol. Psychiatry.

[8] van den Brink A.C., Brouwer-Brolsma E.M., Berendsen A.A.M., van de Rest O. (2019). The Mediterranean, Dietary Approaches to Stop Hypertension (DASH), and Mediterranean-DASH Intervention for Neurodegenerative Delay (MIND) diets are associated with less cognitive decline and a lower risk of Alzheimer’s disease—A review. Adv. Nutr..

[9] Cauli O. (2021). Oxidative stress and cognitive alterations induced by cancer chemotherapy drugs: A scoping review. Antioxidants.

[10] Voynov M., Pospelova M., Nikolaeva A., Krasnikova V., Makhanova A., Fionik O., Samochernykh K., Alekseeva T., Combs S.E., Shevtsov M. (2026). Multimodal cancer therapy and accelerated brain aging: Mechanisms, biomarkers, and clinical consequences. Curr. Oncol..

[11] Ren X., Boriero D., Chaiswing L., Bondada S., St Clair D.K., Butterfield D.A. (2019). Plausible biochemical mechanisms of chemotherapy-induced cognitive impairment (“chemobrain”), a condition that significantly impairs the quality of life of many cancer survivors. Biochim. Biophys. Acta Mol. Basis Dis..

[12] Kim H.G., Rashid M.A., Poleschuk M., Ullah F., Lee S.H., Kim S.H., Qin B., Zheng X.F.S., Jang M.H. (2025). Cognitive dysfunction in chemobrain: Molecular mechanisms and therapeutic implications. Biomed. Pharmacother..

[13] Crowder S.L., Gudenkauf L.M., Hoogland A.I., Han H.S., Small B.J., Carson T.L., Parker N.H., Booth-Jones M., Jim H.S.L. (2025). Cancer-related cognitive impairment and the potential of dietary interventions for the prevention and mitigation of neurodegeneration. Cancer Res..

[14] He Y.Q., Zhou C.C., Jiang S.G., Lan W.Q., Zhang F., Tao X., Chen W.S. (2024). Natural products for the treatment of chemotherapy-related cognitive impairment and prospects of nose-to-brain drug delivery. Front. Pharmacol..

[15] Winschel T.R., Weinhold K., Schnell P.M., Gorka S., Lustberg M., Aase D., Melink Z., Kopec R., Picino C., Li Z. (2025). The association of the MIND diet and its components with cognitive function in postmenopausal breast cancer survivors. Support. Care Cancer.

[16] Zeng Y., Li H., Zi J., Hu Y., Li X., Cao Q., Li Y., Ran Z., Wang X., Cheng G. (2025). Association between dietary oxidative balance score and cognitive function among older adults: A sex- and sex hormone-stratified analysis from the National Health and Nutrition Examination Surveys 2013–2014. J. Affect. Disord..

[17] Cheng X., Cheng L., He J., Wang Y., Lin X., Xia S. (2024). The mediating role of oxidative stress on the association between oxidative balance score and cancer-related cognitive impairment in lung cancer patients: A cross-sectional study. Nutrients.

[18] Carrillo J.Á., Arcusa R., Zafrilla M.P., Marhuenda J. (2021). Effects of fruit and vegetable-based nutraceutical on cognitive function in a healthy population: Placebo-controlled, double-blind, and randomized clinical trial. Antioxidants.

[19] Stavrinou P.S., Andreou E., Aphamis G., Pantzaris M., Ioannou M., Patrikios I.S., Giannaki C.D. (2020). The effects of a 6-month high dose omega-3 and omega-6 polyunsaturated fatty acids and antioxidant vitamins supplementation on cognitive function and functional capacity in older adults with mild cognitive impairment. Nutrients.

[20] Martínez-Lapiscina E.H., Clavero P., Toledo E., Estruch R., Salas-Salvadó J., San Julián B., Sanchez-Tainta A., Ros E., Valls-Pedret C., Martinez-Gonzalez M.Á. (2013). Mediterranean diet improves cognition: The PREDIMED-NAVARRA randomised trial. J. Neurol. Neurosurg. Psychiatry.

[21] Campbell E.K., Campbell T.M., Culakova E., Blanchard L., Wixom N., Guido J.J., Fetten J., Huston A., Shayne M., Janelsins M.C. (2024). A whole food, plant-based randomized controlled trial in metastatic breast cancer: Feasibility, nutrient, and patient-reported outcomes. Breast Cancer Res. Treat..

[22] Lu J., Li D., Li F., Zhou A., Wang F., Zuo X., Jia X.F., Song H., Jia J. (2011). Montreal cognitive assessment in detecting cognitive impairment in Chinese elderly individuals: A population-based study. J. Geriatr. Psychiatry Neurol..

[23] Yang Y. (2018). China Food Composition Tables (Standard Edition).

[24] Zhang Y., Lan X., Li F., Sun H., Zhang J., Li R., Gao Y., Dong H., Cai C., Zeng G. (2022). Dietary cholesterol and egg intake are associated with the risk of gestational diabetes: A prospective study from Southwest China. BMC Pregnancy Childbirth.

[25] Cho A.R., Kwon Y.J., Lee J.H. (2023). Oxidative balance score is inversely associated with the incidence of non-alcoholic fatty liver disease. Clin. Nutr..

[26] Wang H.T., Shou J., Ren L.M., Liu Y. (2019). Reliability and validity of the 12-item short-form health survey questionnaire among the elderly in Shanghai. Chin. Gen. Pract..

[27] Ware J.E., Kosinski M., Turner-Bowker D.M., Gandek B. (2002). How to Score Version 2 of the SF-12 Health Survey (with a Supplement Documenting Version 1).

[28] PDQ® Supportive and Palliative Care Editorial Board Cognitive Impairment in Adults with Cancer (PDQ^®^)—Health Professional Version. https://www.cancer.gov/about-cancer/treatment/side-effects/memory/cognitive-impairment-hp-pdq.

[29] Wei Q., Du B., Liu Y., Cao S., Yin S., Zhang Y., Ye R., Bai T., Wu X., Tian Y. (2024). The Montreal cognitive assessment: Normative data from a large, population-based sample of Chinese healthy adults and validation for detecting vascular cognitive impairment. Front. Neurosci..

[30] Cohen J. (1988). Statistical Power Analysis for the Behavioral Sciences.

[31] Dangour A.D., Allen E., Elbourne D., Fasey N., Fletcher A.E., Hardy P., Holder G.E., Knight R., Letley L., Richards M. (2010). Effect of 2-y n-3 long-chain polyunsaturated fatty acid supplementation on cognitive function in older people: A randomized, double-blind, controlled trial. Am. J. Clin. Nutr..

[32] Kryscio R.J., Abner E.L., Caban-Holt A., Lovell M., Goodman P., Darke A.K., Yee M., Crowley J., Schmitt F.A. (2017). Association of antioxidant supplement use and dementia in the Prevention of Alzheimer’s Disease by Vitamin E and Selenium Trial (PREADViSE). JAMA Neurol..

[33] Zhang Y.P., Miao R., Li Q., Wu T., Ma F. (2017). Effects of DHA supplementation on hippocampal volume and cognitive function in older adults with mild cognitive impairment: A 12-month randomized, double-blind, placebo-controlled trial. J. Alzheimers Dis..

[34] Wang X., Hu J., Liu L., Zhang Y., Dang K., Cheng L., Zhang J., Xu X., Li Y. (2023). Association of dietary inflammatory index and dietary oxidative balance score with all-cause and disease-specific mortality: Findings of 2003–2014 National Health and Nutrition Examination Survey. Nutrients.

[35] Song L., Li H., Fu X., Cen M., Wu J. (2023). Association of the oxidative balance score and cognitive function and the mediating role of oxidative stress: Evidence from the National Health and Nutrition Examination Survey (NHANES) 2011–2014. J. Nutr..

[36] Jin Y., Lin H., Ye Z., Wang H., Liu Y., Qiu W., Liu C. (2025). Associations of oxidative balance score and cognition in US older adults: A cross-sectional study of National Health and Nutrition Examination Survey (NHANES) 2011 to 2014. J. Alzheimers Dis. Rep..

[37] Tönnies E., Trushina E. (2017). Oxidative stress, synaptic dysfunction, and Alzheimer’s disease. J. Alzheimers Dis..

[38] Singh A., Kukreti R., Saso L., Kukreti S. (2019). Oxidative stress: A key modulator in neurodegenerative diseases. Molecules.

[39] Jomova K., Alomar S.Y., Alwasel S.H., Nepovimova E., Kuca K., Valko M. (2024). Several lines of antioxidant defense against oxidative stress: Antioxidant enzymes, nanomaterials with multiple enzyme-mimicking activities, and low-molecular-weight antioxidants. Arch. Toxicol..

[40] Annuzzi G., Bozzetto L., Costabile G., Giacco R., Mangione A., Anniballi G., Vitale M., Vetrani C., Cipriano P., Della Corte G. (2014). Diets naturally rich in polyphenols improve fasting and postprandial dyslipidemia and reduce oxidative stress: A randomized controlled trial. Am. J. Clin. Nutr..

[41] Mier-Cabrera J., Aburto-Soto T., Burrola-Méndez S., Jiménez-Zamudio L., Tolentino M.C., Casanueva E., Hernández-Guerrero C. (2009). Women with endometriosis improved their peripheral antioxidant markers after the application of a high antioxidant diet. Reprod. Biol. Endocrinol..

[42] Pirouzeh R., Heidarzadeh-Esfahani N., Morvaridzadeh M., Izadi A., Yosaee S., Potter E., Heshmati J., Pizarro A.B., Omidi A., Heshmati S. (2020). Effect of DASH diet on oxidative stress parameters: A systematic review and meta-analysis of randomized clinical trials. Diabetes Metab. Syndr..

[43] McKay D.L., Chen C.Y.O., Yeum K.J., Matthan N.R., Lichtenstein A.H., Blumberg J.B. (2010). Chronic and acute effects of walnuts on antioxidant capacity and nutritional status in humans: A randomized, cross-over pilot study. Nutr. J..

[44] Crane T.E., Kubota C., West J.L., Kroggel M.A., Wertheim B.C., Thomson C.A. (2011). Increasing the vegetable intake dose is associated with a rise in plasma carotenoids without modifying oxidative stress or inflammation in overweight or obese postmenopausal women. J. Nutr..

[45] Hayes J.D., Dinkova-Kostova A.T., Tew K.D. (2020). Oxidative stress in cancer. Cancer Cell.

[46] Neth B.J., Schagen S.B., Wefel J.S. (2026). Cancer treatment–related cognitive impairment. Neuro-Oncol. Adv..

[47] Gulcin İ. (2025). Antioxidants: A comprehensive review. Arch. Toxicol..

[48] Wang Y., Liu Y., Cheng L., He J., Cheng X., Lin X., Miao X., Huang Z., Xia S. (2025). Effects of 12-week dietary inflammatory index-based dietary education on frailty status in frail patients with colorectal cancer: A randomized controlled trial. Nutrients.

[49] Yan H., He F., Wei J., Zhang Q., Guo C., Ni J., Yang F., Chen Y. (2023). Effects of individualized dietary counseling on nutritional status and quality of life in post-discharge patients after surgery for gastric cancer: A randomized clinical trial. Front. Oncol..

[50] Von Ah D., Rio C.J., Carter A., Perkins S.M., Stevens E., Rosko A., Davenport A., Kalady M., Noonan A.M., Crouch A. (2024). Association between cognitive function and physical function, frailty, and quality of life in older breast cancer survivors. Cancers.

[51] Ho H.T., Lin S.I., Guo N.W., Yang Y.C., Lin M.H., Wang C.S. (2022). Executive function predict the quality of life and negative emotion in older adults with diabetes: A longitudinal study. Prim. Care Diabetes.

[52] Kazazi L., Shati M., Mortazavi S.S., Nejati V., Foroughan M. (2021). The impact of computer-based cognitive training intervention on the quality of life among elderly people: A randomized clinical trial. Trials.

