# Supplementary material for: A 12-Week Structured Antioxidant-Focused Dietary Intervention Improves Cognitive Function and Oxidative Stress Biomarkers in Lung Cancer Patients with Cancer-Related Cognitive Impairment: A Randomized Controlled Trial
Source: Antioxidants (Basel). 2026 Jul 28;15(8):932. doi: 10.3390/antiox15080932 (PMC13509763; doi:10.3390/antiox15080932)
Supplement: Supplementary file 1 [file antioxidants-15-00932-s001.zip › antioxidants-4426362-supplementary.pdf]

Table S1. Structure and components of the antioxidant-focused dietary intervention

Stage	Setting	Delivery mode	Objective	Core intervention components
Baseline	Pre-intervention	Face-to-face	Baseline assessment and rapport establishment	Obtain informed consent; conduct baseline assessments; perform dietary assessment interviews; involve caregivers in the intervention process.
Stage 1 (Weeks 1–3)	In-hospital	Face-to-face; 30 min	Establish fundamental understanding of antioxidant nutrition and CRCI	Introduce CRCI and its potential relationship with oxidative stress and diet; provide education regarding antioxidant-oriented dietary patterns and common dietary misconceptions; introduce dietary self-monitoring tools and dietary record procedures.<br><br>The education additionally addressed the adverse effects of CRCI, the relationship between diet, oxidative stress, and cognitive function, and the common misconception of relying on single antioxidant supplements. The core dietary targets were:<br>vegetables 300–500 g/day, with ≥50% being dark-colored vegetables (e.g., spinach, broccoli, and carrots); fruits 200–350 g/day, with an emphasis on dark-colored varieties (e.g., blueberries, strawberries, grapes, and kiwis); whole grains 75–150 g/day, accounting for >1/3 of staple foods (e.g., brown rice, black rice, and whole-wheat bread); nuts 15–20 g/day (e.g., walnuts, almonds, cashews, and peanuts); legumes 25 g/day (e.g., approximately 100 g tofu or 400 mL soy milk); fish 300–500 g/week (≥2 times/week); olive, flaxseed, or camellia oil 10 g/day; red meat once/week (≤100 g) and white meat 3–4 times/week (100–120 g/time). Participants were advised to reduce their intake of sugary drinks, refined sugar products, excessive refined carbohydrates, trans fats, and processed meats and to primarily use steaming, boiling, and stewing as cooking methods.<br><br>Dietary self-monitoring tools included kitchen scales and portion-size reference charts, and dietary intake was recorded using written logs, photos, or videos.<br><br>Encourage participants to consume at least one antioxidant-rich food each day.
	Post-discharge	Online (WeChat/telephone)	Initiate dietary behavior change	Provide educational materials for home-based learning; review dietary records; deliver individualized dietary guidance and feedback; provide tailored recommendations of antioxidant-rich foods and cooking strategies; encourage patient engagement and self-monitoring. Encourage participants to interact with the nutrition educator at least twice per week.

Table S1. *Cont.*

Stage	Setting	Delivery mode	Objective	Core intervention components
Stage 2 (Weeks 4–6)	In-hospital	Face-to-face; 30 min	Enhance dietary self-management skills	Assess understanding of antioxidant dietary principles and self-monitoring strategies; reinforce key dietary concepts; assist participants and caregivers in developing individualized dietary plans.
	Post-discharge	Online (WeChat/telephone)	Reinforce adherence to dietary behaviors	Monitor dietary adherence and provide ongoing individualized feedback and behavioral support; encourage continued implementation of antioxidant-oriented dietary practices.
Stage 3 (Weeks 7–9)	In-hospital	Face-to-face; 30 min	Strengthen behavioral reinforcement and problem-solving	Review dietary implementation; identify barriers to adherence; provide tailored guidance according to individual dietary patterns and challenges.
	Post-discharge	Online (WeChat/telephone)	Optimize dietary patterns and maintain engagement	Continue dietary monitoring and individualized support; reinforce healthy dietary behaviors and long-term adherence strategies.
Stage 4 (Weeks 10–12)	In-hospital	Face-to-face; 30 min	Consolidate long-term dietary behavior change	Consolidate antioxidant-oriented dietary knowledge and behaviors; develop individualized strategies for maintaining long-term adherence.
	Post-discharge	Online (WeChat/telephone)	Maintain long-term adherence and intervention effects	Provide continued support and follow-up guidance; reinforce sustainable dietary behaviors and self-management practices.

Table S2. Comparison of baseline characteristics between dropout and completed groups

Variable	Total (n = 100)	Dropout (n = 9)	Completed (n = 91)	t/χ^2	***P***
Age (years) [a]	66.10 ± 5.39	64.67 ± 4.80	66.24 ± 5.45	−0.928	0.375
BMI (kg/m^2) [a]	23.29 ± 2.97	24.23 ± 1.23	23.19 ± 3.08	1.992	0.060
Sex [b]					
Male	78 (78.0)	6 (66.7)	72 (79.1)	0.192	0.661
Female	22 (22.0)	3 (33.3)	19 (20.9)		
Smoking status [c]					
Never	37 (37.0)	4 (44.4)	33 (36.3)		
Former	57 (57.0)	5 (55.6)	52 (57.1)	-	0.852
Current	6 (6.0)	0 (0.0)	6 (6.6)		
Alcohol use [c]					
Never	46 (46.0)	4 (44.4)	42 (46.2)		
Former	51 (51.0)	5 (55.6)	46 (50.5)	-	1.000
Current	3 (3.0)	0 (0.0)	3 (3.3)		
Marital status [b]					
Unmarried/divorced/widowed	2 (2.0)	0 (0.0)	2 (2.2)	0.000	1.000
Married	98 (98.0)	9 (100.0)	89 (97.8)		
Years of education [c]					
No formal education	12 (12.0)	1 (11.1)	11 (12.1)		
1–6 years	30 (30.0)	3 (33.3)	27 (29.7)	-	1.000
≥ 7 years	58 (58.0)	5 (55.6)	53 (58.2)		
Employment [c]					
Employed	1 (1.0)	0 (0.0)	1 (1.1)		
Unemployed	6 (6.0)	0 (0.0)	6 (6.6)	-	1.000
Retired	93 (93.0)	9 (100.0)	84 (92.3)		
Residence [c]					
Rural areas	26 (26.0)	5 (55.6)	21 (23.1)		
Towns	14 (14.0)	0 (0.0)	14 (15.4)	-	0.092
Urban areas	60 (60.0)	4 (44.4)	56 (61.5)		
Family monthly income (CNY) [c]					
< 3000	15 (15.0)	0 (0.0)	15 (16.5)		
3000–5000	39 (39.0)	4 (44.4)	35 (38.5)	-	0.575
> 5000	46 (46.0)	5 (55.6)	41 (45.1)		
Physical activity level [c]					
Low	30 (30.0)	4 (44.4)	26 (28.6)		
Moderate	63 (63.0)	5 (55.6)	58 (63.7)	-	0.515
High	7 (7.0)	0 (0.0)	7 (7.7)		
Presence of comorbidities [b]					
No	50 (50.0)	4 (44.4)	46 (50.5)	0.000	1.000
Yes	50 (50.0)	5 (55.6)	45 (49.5)		
Pathology subtype [b]					
Non-small cell lung cancer	91 (91.0)	9 (100.0)	82 (90.1)	0.143	0.705
Small cell lung cancer	9 (9.0)	0 (0.0)	9 (9.9)		

Table S2. *Cont.*

Variable	Total (n = 100)	Dropout (n = 9)	Completed (n = 91)	t/χ^2	***P***
Cancer stage [c]					
I	18 (18.0)	1 (11.1)	17 (18.7)	-	0.849
II	12 (12.0)	1 (11.1)	11 (12.1)		
III	31 (31.0)	2 (22.2)	29 (31.9)		
IV	39 (39.0)	5 (55.6)	34 (37.4)		
Treatment modality [c]					
Chemotherapy alone	20 (20.0)	2 (22.2)	18 (19.8)	-	1.000
Chemotherapy plus immunotherapy	26 (26.0)	2 (22.2)	24 (26.4)		
Targeted therapy-containing regimens	27 (27.0)	2 (22.2)	25 (27.5)		
Other systemic therapy	27 (27.0)	3 (33.3)	24 (26.4)		
History of surgery [b]					
No	57 (57.0)	5 (55.6)	52 (57.1)	0.000	1.000
Yes	43 (43.0)	4 (44.4)	39 (42.9)		

Data are presented as *n* (%) or mean ± SD. [a] Independent-samples t-test. [b] Chi-squared test with continuity correction.

[c] Fisher's exact test. CNY, Chinese yuan.

Table S3. Sensitivity analyses of intervention effects after additional adjustment for baseline BMI

Outcome domain	Variable	*β* (95% CI)	*P*
Cognitive function	MoCA	2.624 (0.821, 4.426)	0.005
Dietary oxidative balance	DOBS	2.293 (0.652, 3.934)	0.006
Oxidative stress biomarkers	GSH	0.106 (0.032, 0.179)	0.006
	SOD	0.055 (0.004, 0.106)	0.035
	MDA	−0.164 (−0.267, −0.062)	0.002
	CAT	0.007 (−0.090, 0.103)	0.891
	GPx	0.001 (−0.026, 0.029)	0.925
Quality of life	PCS	1.368 (0.029, 2.708)	0.045
	MCS	3.398 (0.955, 5.842)	0.007

DOBS: dietary oxidative balance score. MoCA: Montreal Cognitive Assessment. PCS: Physical Component Summary. MCS: Mental Component Summary. GSH: glutathione. SOD: superoxide dismutase. MDA: malondialdehyde. CAT: catalase. GPx: glutathione peroxidase. Adjusted for age, sex, years of education, cancer stage, treatment modality and baseline BMI.

Table S4. Descriptive summary of DOBS and daily nutrient intakes at baseline and week 12

Variable	Intervention		Control	
	**Baseline (*n* = 50)**	**Week 12 (*n* = 45)**	**Baseline (*n* = 50)**	**Week 12 (*n* = 46)**
DOBS	12.44 ± 2.98	13.60 ± 2.52	12.24 ± 3.30	11.09 ± 2.61
MUFAs (g/d)	14.27 (10.88, 16.25)	16.13 (12.70, 19.17)	13.76 (10.69, 24.82)	12.76 (10.15, 16.48)
SFAs (g/d)	14.15 (8.34, 15.82)	12.27 (6.99, 16.62)	13.48 (8.10, 18.70)	15.29 (12.24, 21.74)
n-3 PUFAs (g/d)	1.33 (1.00, 1.56)	1.54 (1.18, 3.44)	1.29 (1.09, 1.56)	1.27 (1.15, 1.60)
n-6 PUFAs (g/d)	8.03 (4.78, 9.86)	8.19 (6.58, 9.96)	8.29 (6.76, 9.94)	8.86 (7.41, 10.80)
β-carotene (μg/d)	1025.35 (483.54, 1977.92)	1263.41 (498.87, 2949.24)	1085.12 (542.83, 1539.76)	1125.46 (467.96, 1580.93)
Selenium (μg/d)	41.61 (25.02, 49.23)	41.80 (26.72, 47.98)	41.69 (28.33, 56.98)	41.22 (24.14, 53.95)
Vitamin A (μgRAE/d)	386.99 (215.01, 419.47)	391.39 (227.19, 450.97)	369.23 (197.66, 431.65)	362.06 (200.54, 415.31)
Vitamin C (mg/d)	77.45 (41.14, 99.64)	92.11 (41.74, 152.89)	81.20 (42.10, 93.14)	55.39 (44.78, 92.15)
Vitamin E (mg/d)	10.46 (5.49, 18.61)	12.94 (5.81, 18.67)	10.25 (7.74, 15.84)	10.32 (5.98, 14.08)
Dietary fiber (g/d)	6.15 (4.66, 12.51)	7.81 (5.80, 12.80)	6.11 (5.23, 11.64)	6.05 (4.94, 7.21)
Iron (mg/d)	12.85 (9.89, 21.98)	12.50 (10.30, 19.57)	12.56 (7.08, 19.06)	12.54 (9.15, 19.56)
Zinc (mg/d)	8.09 (6.39, 10.39)	8.24 (6.57, 9.59)	8.02 (6.57, 10.52)	8.10 (6.84, 10.49)

Data are presented as mean ± standard deviation (SD) for DOBS and as median (Q1, Q3) for nutrient intakes. DOBS: dietary oxidative balance score. SFAs: saturated fatty acids. MUFAs: monounsaturated fatty acids. n-3 PUFAs: n-3 polyunsaturated fatty acids. n-3 PUFAs: n-6 polyunsaturated fatty acids.

Table S5. Descriptive summary of oxidative stress biomarkers at baseline and at week 12

Variable	Intervention		Control	
	**Baseline (*n* = 50)**	**Week 12 (*n* = 44)**	**Baseline (*n* = 50)**	**Week 12 (*n* = 44)**
GSH (μmol/L)	3.66 (2.99, 4.19)	4.32 (3.56, 5.59)	3.36 (2.88, 4.47)	3.35 (2.98, 4.09)
SOD (U/mL)	31.95 (25.35, 36.92)	34.19 (29.02, 38.58)	32.96 (27.69, 37.04)	32.52 (23.48, 36.60)
MDA (nmol/mL)	2.94 (2.15, 4.01)	1.95 (1.55, 3.03)	2.75 (2.23, 3.79)	3.25 (2.43, 4.23)
CAT (U/mL)	7.80 (5.91, 9.13)	8.03 (6.35, 9.83)	7.11 (5.04, 9.51)	6.95 (5.60, 8.31)
GPx (U/mL)	181.08 (161.08, 196.90)	185.35 (164.79, 204.13)	183.78 (159.93, 201.35)	181.88 (163.51, 190.14)

Data are presented as median (Q1, Q3). GSH: glutathione. SOD: superoxide dismutase. MDA: malondialdehyde. CAT: catalase. GPx: glutathione peroxidase.

Table S6. Descriptive summary of quality of life at baseline and at week 12

Variable	Intervention (*n* = 50)		Control (*n* = 50)	
	Baseline (*n* = 50)	Week 12 (*n* = 45)	Baseline (*n* = 50)	Week 12 (*n* = 46)
PCS	38.35 ± 7.84	39.45 ± 5.16	38.45 ± 8.58	38.55 ± 8.45
MCS	39.34 ± 7.21	42.72 ± 4.40	39.50 ± 7.64	39.61 ± 7.91

Data are presented as mean ± standard deviation (SD). PCS: Physical Component Summary. MCS: Mental Component Summary.
